# The Interplay Between CB_2_ and NMDA Receptors in Parkinson’s Disease

**DOI:** 10.3390/ijms26199419

**Published:** 2025-09-26

**Authors:** Irene Reyes-Resina, Jaume Lillo, Iu Raïch, Joan Biel Rebassa, Toni Capó, Pau Badia, Gemma Navarro

**Affiliations:** 1 Department of Biochemistry and Physiology, School of Pharmacy and Food Sciences, University of Barcelona, 08028 Barcelona, Spain; jaumelillo@ub.edu (J.L.); iraichpa7@ub.edu (I.R.); jrebaspa7@alumnes.ub.edu (J.B.R.); tonicapoquetglas@ub.edu (T.C.); pbadiafe47@alumnes.ub.edu (P.B.); 2 Network Center for Biomedical Research in Neurodegenerative Diseases, CiberNed, Spanish National Health Institute Carlos III, Av. Monforte de Lemos, 3-5, 28029 Madrid, Spain; 3 Institut de Neurociències UB, Campus Mundet, Passeig de la Vall d’Hebron 171, 08035 Barcelona, Spain

**Keywords:** cannabinoid receptor 2, NMDA receptor, Parkinson’s disease, α-synuclein, receptor heteromer

## Abstract

Parkinson’s disease (PD) is a progressive neurological disorder that affects movement, causing symptoms such as tremors, stiffness, slowness, and balance problems due to the degeneration of dopamine-producing neurons in the brain. Nowadays there is no cure for PD. Alpha synuclein (α-syn) aggregates, which are a hallmark of PD, are known to induce microglial activation, specifically the detrimental M1 microglial phenotype, which contributes to neuroinflammation and disease progression. Cannabinoid receptor 2 (CB_2_R) activation has been shown to counteract neuroinflammation. CB_2_R is able to interact with N-methyl-D-aspartate (NMDA) receptors (NMDAR), which has also attracted attention in PD research due to its role in excitotoxicity. Here we aimed to study the interaction between CB_2_R and NMDAR in a PD context in rat tissue. We observed that α-syn fibrils alter CB_2_R activation and CB_2_R-NMDAR heteromerization in a heterologous expression system. Furthermore, activation of CB_2_R counteracted NMDAR signaling. In microglia, α-syn fibrils decreased CB_2_R-NMDAR heteromer expression while increasing CB_2_R signaling. Importantly, CB_2_R activation counteracted the α-syn fibrils-induced increase in M1-activated microglia, while it favored the polarization of microglia to the beneficial M2 phenotype. These results reinforce the idea of using cannabinoids for treating PD, as they provide not only the anti-inflammatory effects of cannabinoids but also counteract the detrimental increase in NMDAR signaling present in this disease.

## 1. Introduction

Parkinson’s disease is the most common neurodegenerative movement disorder [1]. In Europe, prevalence and incidence rates for PD are estimated at approximately 200/100,000 and 15/100,000 per year, respectively [2]. Age and male gender are considered risk factors [3]. This pathology is characterized by the loss of the dopaminergic neurons of the substantia nigra pars compacta, whose axons project to the striatum. As a consequence of the lack of dopamine in the striatum, the direct and indirect pathways are dysregulated, and symptoms such as bradykinesia, rigidity, tremor, and postural instability appear [4]. The etiology of the disease in most patients is unknown. The familial form of Parkinson’s disease accounts for only 14% of all cases, and it has been linked to mutations in different genes, such as alpha-synuclein (α-syn), parkin, DJ-1, PINK1, and LRRK2 [5]. The majority of PD cases are sporadic, being associated with variants of genes that can influence the susceptibility to PD, which have been suggested to be caused by interaction with environmental toxins [6].

PD is characterized by the presence of Lewy bodies, which are intracellular inclusions present in the remaining dopaminergic neurons, composed of aggregates of misfolded α-synuclein [7]. This presynaptic protein seems to have a role in the biosynthesis and liberation of neurotransmitter vesicles by promoting SNARE-complex assembly during vesicle docking and fusion steps [8]. In both PD forms, the presence of α-syn aggregates has been described. The misfolded form of α-syn, that appears due to mutations or polymorphisms in the SNCA gene [9,10], aggregates forming oligomers, which will further aggregate in the form of fibrils that finally generate Lewy bodies [11]. Current treatment for PD is based, among others, on the replacement of dopamine, with the most common treatment being the dopamine precursor levodopa (L-DOPA), which produces secondary effects such as the abnormal involuntary movements known as dyskinesias [12]. Alternative approaches such as deep brain stimulation (DBS) are suitable for later-stage disease. Current available treatments offer good control of motor symptoms but do not halt the progression of neurodegeneration, the evolution of the disease, and the increasing disability.

Cumulative evidence points to the role of α-synuclein in activating immunological response, as it has been described that Lewy body-like alpha-synuclein inclusions trigger reactive microgliosis prior to nigral degeneration [13]. Furthermore, it has been demonstrated that activated microglial cells directly engulf α-synuclein in an attempt to clear it [14]. Indeed, it is known that neurodegenerative diseases such as PD or AD present neuroinflammation. Post-mortem analyses in PD patients revealed the presence of activated microglia and increased expression of inflammatory markers in the substantia nigra, putamen, and cortex [15,16]. Analysis of PD patients’ post-mortem brain tissue revealed a correlation between microglial activation and α-synuclein pathology in the SN and the hippocampus [15]. A recent study proposes a model in which α-syn accumulation activates the detrimental M1 type microglia, while it does not activate the M2 type, which is considered neuroprotective [17].

Cannabinoids have been proposed to decrease neuroinflammation in neurodegenerative diseases [18,19]. Cannabinoids mainly act on cannabinoid CB_1_R and CB_2_R receptors, with CB_2_R being of great interest, as its activation lacks psychoactive effects [20]. CB_2_R expression is higher in glial cells than in neurons, and it increases upon microglial activation, which seems to underlie the neuroprotective effects of cannabinoids [21,22]. Furthermore, an increase in CB_2_R expression was found in microglial cells in the post-mortem substantia nigra of PD patients and in the striatum and the substantia nigra in lipopolysaccharide (LPS)-lesioned mice [23]. The 1-methyl-4-phenyl-1,2,3,6-tetrahydropyridine (MPTP) neurotoxic mouse PD model shows a significant microglial activation and overexpression of CB_2_ receptors in the midbrain [24]. This increase in CB_2_R expression was also found in the neurotoxic 6-hydroxydopamine (6-OHDA), the environmental and the inflammation-driven rat models of Parkinson’s disease, and this change significantly correlated with an increase in microglial activation [25,26]. The pharmacological activation of CB_2_ receptors has been shown to reduce proinflammatory responses in the LPS-induced mouse model [23], in the MPTP neurotoxic mouse models [24], and in the rotenone rat model [27] of PD, and thus to protect dopaminergic neurons from degeneration. On the contrary, the genetic inactivation of CB_2_ receptors aggravated LPS-induced inflammation [23] and led to a more pronounced MPTP-induced toxicity in the substantia nigra [24], respectively. Hence, this receptor has been proposed as a therapeutic target for Parkinson’s disease [23,28].

The ability of G protein-coupled receptors (GPCRs) to form complexes with other membrane receptors is well known [29]. Cannabinoid receptors CB_1_ and CB_2_ have been described to form complexes, among others, with NMDA receptors in the central nervous system, both in neurons and in glia [30,31]. CB_1_R-NMDAR complexes seem to play an important role in Parkinson’s [31] disease; however, the role of CB_2_R-NMDAR complexes in a Parkinson’s disease context remains unexplored [30].

Furthermore, the alteration in the expression and function of NMDARs is one of the hallmarks of PD. NMDARs are heteromeric glutamate-gated ion channels formed by four subunits, which in humans are usually two obligatory NR1 subunits plus two NR2A or NR2B subunits [32]. They are implicated in synaptic plasticity, learning, and memory, but also in neurodegeneration and excitotoxicity [33,34,35]. In the rat PD model consisting of unilateral nigrostriatal dopamine system ablation with 6-OHDA, the NMDAR phosphorylation state is altered [36,37]. Also, in PD patients and in animal models of the disease, an alteration in the expression levels of NMDAR subunits has been found [36,38,39]. Interestingly, NMDAR antagonists such as MK-801, or negative allosteric modulators of NMDAR such as radiprodil, have been shown to improve motor symptoms in PD [40,41]. However, there is currently no cure for Parkinson’s disease, only symptomatic treatments are available, often with undesired secondary effects [42].

Given the lack of a cure for Parkinson’s disease, the aim of the present study was to determine if CB_2_R-NMDAR complexes expressed in microglia could have a role as therapeutic targets in PD. We studied the expression and function of these complexes in a heterologous expression system and in an in vitro model of PD consisting of primary cultures of rat microglia treated with α-syn.

## 2. Results

### 2.1. α-Syn Fibrils Alter the Conformation of CB_2_R-NMDAR Complexes

**Figure 1:**
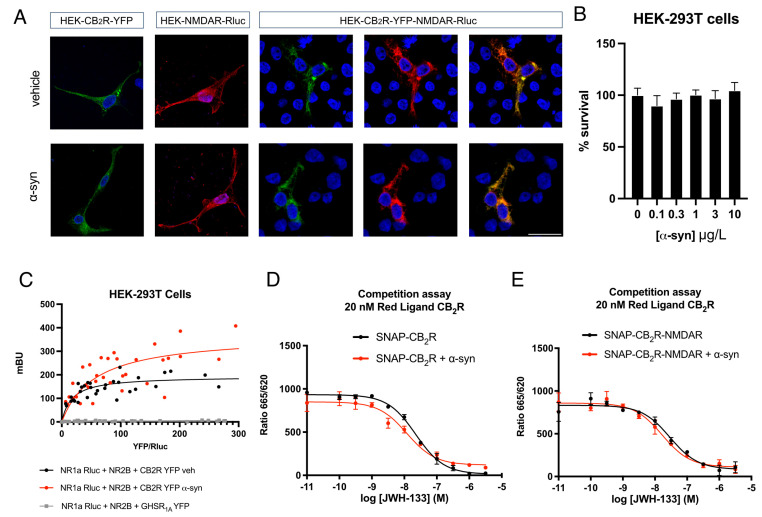
Analysis of CB_2_R-NMDAR complex formation in the presence of α-syn. (**A**) Immunocytochemistry assay was performed in HEK-293T cells treated or not with 10 µg/L of human α-syn fibrils for 48 h and transfected with either CB_2_R-YFP (1 µg cDNA) (shown in green), NR1-Rluc (0.75 µg cDNA) (shown in red) plus NR2B (0.75 µg cDNA), or both. Nuclei were stained with Hoechst (blue). Colocalization is shown in yellow. Scale bar: 20 µm. (**B**) HEK-293T cells were treated with increasing concentrations of α-syn for 48 h. Then, cells were gently detached, and cell survival was assessed with a cell counter. % vs. 0 µg/L α-syn is shown. Values are the mean ± SEM of five independent experiments performed in triplicates. One-way ANOVA, followed by Tukey’s multiple comparison post hoc test, was used for statistical analysis. (**C**) BRET assays were performed in HEK-293T cells treated or not with 10 µg/L of human α-syn fibrils for 48 h and transfected with constant amounts of cDNAs for NR1-Rluc (0.5 µg) and NR2B (0.3 µg) and increasing amounts of cDNA for CB_2_R-YFP (0 to 2.5 µg) or GHSR_1A_-YFP (0 to 10 µg). Values are the mean ± SEM of five different experiments performed in triplicates. (**D**,**E**) HTRF-based competitive binding assays were performed in HEK-293T cells transiently transfected with 1.5 µg cDNA for SNAP-CB_2_R in the absence (**D**) or presence of 0.7 µg cDNA for NR1 and 0.7 µg cDNA for NR2B (**E**). Competition curves of specific binding of 20 nM fluorophore-conjugated CM157 using JWH-133 as competitor are shown. Data represent the mean ± SEM of five experiments performed in triplicates.

To determine if CB_2_R-NMDAR complexes are altered in a Parkinsonian context, we first studied if preformed α-syn fibrils could alter the localization of CB_2_R and NMDAR in the plasma membrane. Human Embryonic Kidney 293T (HEK-293T) cells have been previously described to be able to uptake these α-syn preformed fibrils after a 2-day treatment [31]. HEK-293T cells expressing either CB_2_R fused to the yellow fluorescent protein (CB_2_R-YFP), NMDAR fused to the Renilla luciferase (NMDAR–Rluc), or both constructs, were analyzed under the confocal microscope. CB_2_R expression was detected by the YFP’s own fluorescence, while expression of NMDAR was detected by immunocytochemistry with an anti-Rluc antibody plus a Cy3 conjugated secondary antibody. The expression of both receptors was shown at the plasma membrane level of HEK-293T cells and also in the cytoplasm, and when cells were co-expressing both receptors, colocalization was observed (Figure 1A). When the same experiment was performed in the presence of α-syn fibrils, no differences were found compared to the control condition, neither in the expression nor in the colocalization between CB_2_R and NMDAR (Figure 1A). To rule out the possibility that α-syn fibrils cause HEK-293T cell death, cells were incubated with increasing concentrations of α-syn fibrils for 48 h. When HEK-293T cell viability was measured, we observed that no cell death was caused by α-syn fibrils (Figure 1B).

Next, we aimed to study if α-syn fibrils could alter the formation of CB_2_R-NMDAR complexes. Thus, the Bioluminiscence Resonance Energy Transfer (BRET) technique was carried out to assay physical interactions between receptors in vivo. When we performed a BRET assay in HEK-293T cells expressing constant amounts of NMDAR fused to Rluc and increasing amounts of CB_2_R fused to YFP, a saturation curve was obtained (Figure 1C), which indicates that CB_2_R and NMDAR can physically interact, as previously described [30]. The obtained BRET_max_ was 200 ± 27 mBU (milli BRET units), and the BRET_50_ was 17 ± 10. However, when the same assay was performed in the presence of α-syn fibrils, a higher BRET signal was observed (Figure 1C), with a BRET_max_ of 370 ± 90 mBU and a BRET_50_ of 56 ± 40. These results demonstrate that α-syn fibrils do not disrupt the formation of CB_2_R-NMDAR complexes. Instead, fibrils likely cause a protein reorganization at the plasma membrane that changes the orientation of the Rluc and YFP, resulting in a better energy transfer between them, as described for CB_1_R-NMDAR complexes [31]. To show that the interaction between CB_2_R and NMDAR is specific, a negative control experiment was performed, in which CB_2_R-YFP was substituted by the ghrelin receptor GHSR_1A_ fused to YFP, which resulted in a non-saturable linear signal (Figure 1C), indicative of a lack of interaction between NMDAR and GHSR_1A_.

### 2.2. α-Syn Fibrils Do Not Affect CB_2_R Affinity for Its Ligands

A common phenomenon in GPCR biology is the fact that the interaction with other receptors can alter GPCRs properties, such as ontogeny, ligand binding, pharmacology, signaling, or internalization [43,44,45]. Thus, we first aimed to determine if the interaction of CB_2_R with NMDAR could alter ligand binding to the orthosteric site of CB_2_R. Homogeneous Time-Resolved FRET (HTRF) assays were performed in HEK-293T cells expressing either only CB_2_R fused to the SNAP protein (SNAP-CB_2_R) or SNAP-CB_2_R together with NMDAR, and treated with α-syn fibrils or the vehicle for 48 h. Control cells were treated with increasing amounts of the selective CB_2_R agonist JWH-133 (from 0.1 nM to 10 μM) to displace the fluorophore-conjugated selective CB_2_R agonist CM157. JWH-133 decreased the binding of labeled CM157 to SNAP-CB_2_R in a monophasic fashion in cells expressing only CB_2_R and also in cells expressing CB_2_R-NMDAR (Ki values were 18.0 and 23.4 nM, respectively). These results indicate that the interaction of CB_2_R with NMDAR does not significantly alter the affinity of CB_2_R for JWH-133 (Figure 1D,E). Next, we sought to determine how α-syn fibrils affected the binding of ligands to CB_2_R. When the same HTRF assays were performed in cells treated with α-syn fibrils, monophasic competition curves were obtained again (Figure 1D,E), with Ki values of 9.63 nM for cells expressing SNAP-CB_2_R and 13.5 nM for cells expressing SNAP-CB_2_R plus NMDAR. These results indicate that α-syn fibrils do not affect the affinity of CB_2_R for its selective agonist JWH-133.

### 2.3. CB_2_R Signaling Is Decreased upon Treatment with α-Syn Fibrils

**Figure 2:**
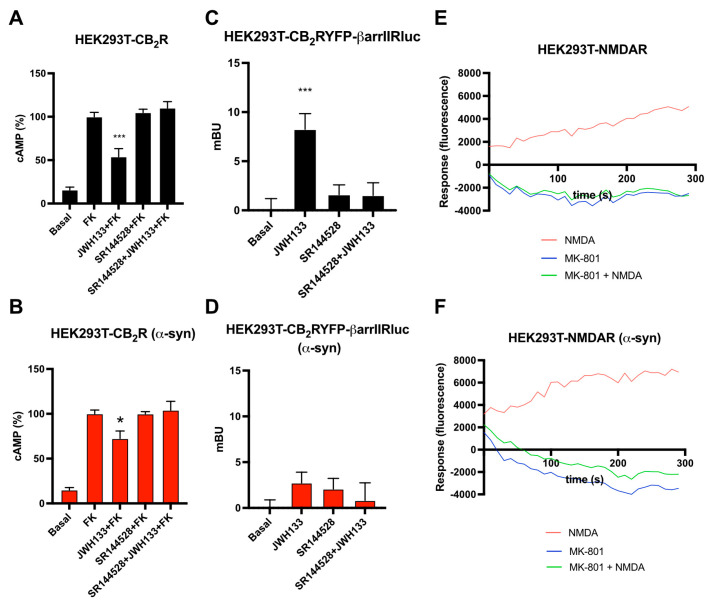
Analysis of CB_2_R and NMDAR signaling in the presence of α-syn fibrils in a heterologous system. (**A**,**B**) HEK-293T cells transfected with CB_2_R (1.3 µg cDNA) were treated (**B**) or not (**A**) with α-syn fibrils. Forty-eight hours after, cells were pre-treated with the vehicle or with the selective CB_2_R antagonists SR144528 1 µM, followed by CB_2_R agonist stimulation (100 nM JWH-133). cAMP accumulation was detected by HTRF in the presence of 0.5 µM forskolin. Values are the mean ± SEM of six different experiments performed in triplicates, and one-way ANOVA, followed by Tukey’s multiple comparison post hoc test, which was used for statistical analysis (* *p* < 0.05, *** *p* < 0.001; versus treatment with forskolin). (**C**,**D**) HEK-293T cells transfected with CB_2_R-YFP (1.2 µg cDNA) together with β-arrestin II-Rluc (0.4 µg cDNA) were treated (**D**) or not (**C**) with α-syn fibrils. Forty-eight hours after, cells were pre-treated with the vehicle or with the selective CB_2_R antagonists SR144528 1 µM, followed by agonist stimulation (100 nM JWH-133). β-arrestin II recruitment was assayed by BRET assays. Values are the mean ± SEM of six different experiments performed in triplicates, and one-way ANOVA, followed by Tukey’s multiple comparison post hoc test, which was used for statistical analysis (*** *p* < 0.001; versus basal). (**E**,**F**) Calcium release was evaluated in HEK-293T transfected with NR1 (1 µg cDNA), NR2B (1 µg cDNA) and with 6GCamMP calcium sensor (0.3 µg cDNA) and treated (**F**) or not (**E**) with α-syn fibrils. Cells were pre-treated with the vehicle or with the selective antagonists (1 µM MK-801), followed by agonist stimulation (15 µM NMDA). Data represent the mean ± SEM of six different experiments performed in triplicates.

After studying how α-syn fibrils affect the formation of CB_2_R-NMDAR heteromers in HEK-293T cells, we wondered how fibrils could affect the functionality of these receptor complexes in this heterologous expression system. Firstly, we determined the effect of α-syn fibrils in the signaling of individual CB_2_R and NMDAR. As CB_2_R is a GPCR coupled with a Gi protein, intracellular cAMP levels were assayed. In HEK-293T cells expressing CB_2_R, the selective CB_2_R agonist JWH-133 was able to induce a 50% decrease in intracellular cAMP levels previously increased by pre-treatment with forskolin (Figure 2A). When these cells were pre-treated with the CB_2_R antagonist SR144528, the effect of JWH133 was blocked (Figure 2A). However, when cAMP levels were tested after incubation with α-syn fibrils, the decrease in cAMP levels induced by JWH-133 was only 30% (Figure 2B). The recruitment of β-arrestin II upon CB_2_R activation was also assayed. A BRET assay was performed in HEK-293T cells expressing CB_2_R fused to YFP and β-arrestin II fused to Rluc. CB_2_R agonist JWH-133 was able to induce a significant β-arrestin II recruitment, which was counteracted by CB_2_R antagonist SR144528 (Figure 2C). The signal induced by JWH-133 was abolished upon α-syn fibrils treatment (Figure 2D). These results indicate that α-syn fibrils reduce CB_2_R activation in HEK-293T cells, in line with the effects caused by α-syn fibrils on CB_1_R signaling [31].

Regarding NMDAR, ion channel activation has no effect either in cAMP levels or in β-arrestin II recruitment, hence intracellular Ca^2+^ mobilization was studied. When HEK-293T cells expressing NMDAR were treated with the NMDAR agonist NMDA, a calcium signal was observed that was counteracted by pre-treatment with the NMDAR antagonist MK-801 (Figure 2E). When calcium mobilization was studied in the presence of α-syn fibrils, no difference was observed compared to cells treated with the vehicle (Figure 2F), indicating that α-syn fibrils do not affect intracellular Ca^2+^ mobilization upon NMDAR activation in HEK-293T cells.

### 2.4. Signaling of CB_2_R-NMDAR Complexes Is Decreased upon α-Syn Fibrils Treatment in a Heterologous System

**Figure 3:**
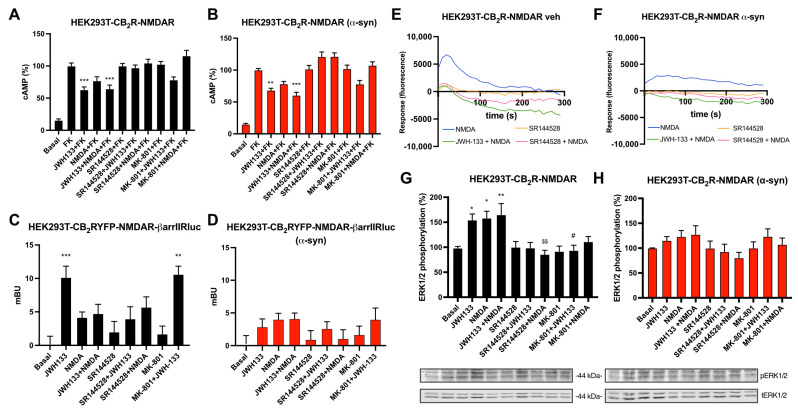
Functional analysis of CB_2_R-NMDAR complexes in a heterologous system upon α-syn treatment. (**A**,**B**,**G**,**H**) HEK-293T cells were transfected with the cDNAs for CB_2_R (1 µg), NR1 (0.7 µg), and NR2B (0.7 µg) and treated (**B**,**H**) or not (**A**,**G**) with α-syn fibrils. Cells were pre-treated with the selective antagonists (1 µM SR144528 for CB_2_R or 1 µM MK-801 for NMDAR), followed by agonist stimulation (100 nM JWH-133 for CB_2_R and/or 15 µM NMDA for NMDAR). cAMP accumulation was detected by HTRF in the presence of 0.5 µM forskolin (**A**,**B**). MAPK phosphorylation was detected by Western blot (blots for phosphorylated and total ERK1/2 (42–44 kDa) are shown below) (**G**,**H**). Values are the mean ± SEM of six different experiments performed in triplicates, and one-way ANOVA, followed by Tukey’s multiple comparison post hoc test, which was used for statistical analysis (* *p* < 0.05, ** *p* < 0.01, *** *p* < 0.001; versus treatment with forskolin in cAMP or versus basal in MAPK, ^#^ *p* < 0.05 versus JWH-133, ^$$^ *p* < 0.01 versus NMDA). (**C**,**D**) HEK-293T cells transfected with CB_2_R-YFP (1.2 µg cDNA), NR1 (0.7 µg), and NR2B (0.7 µg) together with β-arrestin II-Rluc (0.4 µg cDNA) were treated (**D**) or not (**C**) with α-syn fibrils. Forty-eight hours after, cells were pre-treated with the selective antagonists, followed by agonist stimulation. β-arrestin II recruitment was assayed by BRET assays. Values are the mean ± SEM of six different experiments performed in triplicates, and one-way ANOVA, followed by Tukey’s multiple comparison post hoc test, which was used for statistical analysis (** *p* < 0.01, *** *p* < 0.001; versus basal). (**E**,**F**) Calcium release was evaluated in HEK-293T cells transfected with the cDNAs for CB_2_R (1 µg), NR1 (0.7 µg) NR2B (0.7 µg), and 6GCamMP calcium sensor (0.3 µg) and treated (**F**) or not (**E**) with α-syn fibrils. Cells were pre-treated with the selective antagonists, followed by agonist stimulation. Data represent the mean ± SEM of six different experiments performed in triplicates.

After studying the signaling on individual receptors, the effect of α-syn fibrils in the functionality of CB_2_R-NMDAR complexes was tested. Intracellular cAMP levels were assayed in HEK-293T cells expressing both CB_2_R and NMDAR to study the Gi activation pathway. In these cells, the CB_2_R agonist JWH-133 induced a 40% decrease in cAMP levels previously increased by forskolin, while NMDAR agonist did not induce a significant effect, as expected (Figure 3A). When cells were co-treated with both agonists together, a similar effect to that observed with JWH-133 alone was detected, indicating that NMDAR activation does not affect CB_2_R signaling (Figure 3A). JWH-133 signal was blocked by CB2R selective antagonist SR144528, but also by NMDAR antagonist MK-801, as in this condition, JWH-133 was not able to induce a significant decrease in cAMP levels, indicating that there is a cross-antagonism effect from NMDAR to CB_2_R. When cAMP levels were measured after incubation with α-syn fibrils, the signal induced by JWH-133 was similar to that obtained in the absence of fibrils, indicating that in CB_2_R-NMDAR complexes α-syn fibrils do not affect CB_2_R signaling in the intracellular cAMP pathway (Figure 3B). The cross-antagonism effect was also detected.

β-arrestin II recruitment to CB_2_R was studied in HEK-293T cells expressing CB_2_R fused to YFP together with NMDAR, and β-arrestin II fused to Rluc. Stimulation with CB_2_R agonist JWH-133 yielded a significant signal, while, as expected, NMDA treatment induced no response (Figure 3C). Co-treatment with both JWH-133 and NMDAR abolished JWH-133-induced signal (Figure 3C), indicating that NMDAR activation decreases the recruitment of β-arrestin II by CB_2_R. Upon α-syn fibrils treatment, JWH-133 was not able to induce a significant β-arrestin recruitment (Figure 3D), indicating that in this pathway, α-syn fibrils disrupt CB_2_R signaling.

To gain insights into NMDAR signaling, intracellular Ca^2+^ mobilization assays were performed. HEK-293T cells were transfected with CB_2_R, NMDAR, and a calcium sensor protein that emits green fluorescence upon cytoplasmic Ca^2+^ rise [46]. These cells responded to NMDA stimulation, and this signal was significantly decreased by co-treatment with JWH-133, which indicates that CB_2_R activation decreases NMDAR signal (Figure 3E). NMDA-induced signal was counteracted by CB_2_R antagonist SR144528 (Figure 3E), pointing to a cross-antagonism effect from CB_2_R to NMDAR in CB_2_R-NMDAR complexes in HEK-293T cells. Treatment with α-syn fibrils induced a decrease in NMDAR signaling (Figure 3F). These results are similar to those observed in CB_1_R-NMDAR complexes upon α-syn fibrils treatment [31].

A signaling pathway involved in both GPCR and NMDAR activation is the MAP kinase (MAPK) phosphorylation pathway. When HEK-293T cells expressing CB_2_R and NMDAR were stimulated with either the selective CB_2_R agonist JWH-133 or with NMDA, a significant increase in ERK1/2 phosphorylation was detected (Figure 3G). When cells were treated with both agonists together, the signal was smaller than the signal expected after the addition of the individual signals, which is known as non-additive effect (Figure 3G). Pre-treatment with either CB_2_R antagonist SR144528 or with NMDAR antagonist MK-801 blocked both JWH-133- and NMDA-induced signals (Figure 3G), indicating that there is a bidirectional cross-antagonism phenomenon in the MAPK signaling pathway. These results show that in MAPK activation, antagonization of CB_2_R is able to block NMDA-induced signal, and also NMDAR antagonization is able to block JWJ-133-induced signal. When ERK1/2 phosphorylation was tested in the presence of α-syn fibrils, no significant signals were obtained with any of the agonists (Figure 3H), thus fibril treatment abolishes both CB_2_R- and NMDAR-mediated MAPK activation in HEK-293T cells.

### 2.5. The Expression and Function of CB_2_R-NMDAR Complexes Are Altered by α-Syn Fibrils in Rat Microglia

**Figure 4:**
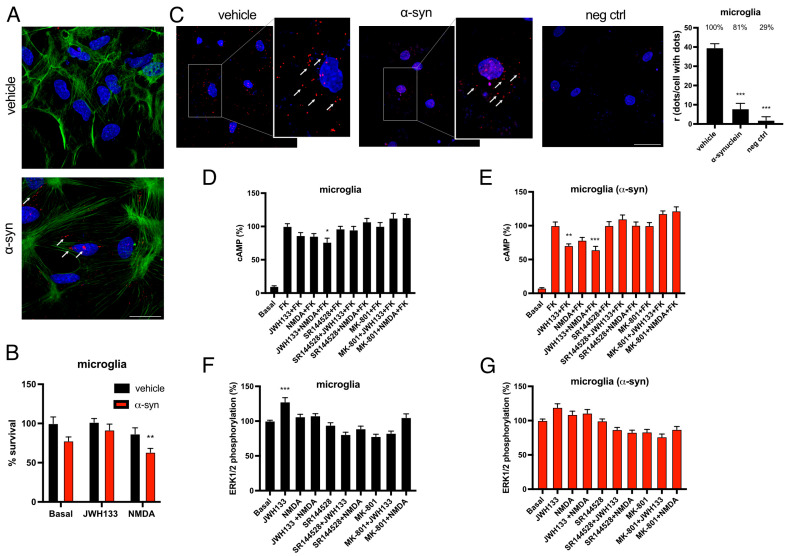
Expression and functionality of CB_2_R-NMDAR complexes in microglia treated with α-syn fibrils. (**A**) Primary cultures of microglia were treated with α-syn or vehicle. α-syn fibrils were detected by immunocytochemistry with a mouse anti-human α-synuclein antibody (red). White arrows indicate α-syn presence. Phalloidin was detected with an AF488-prestained anti-phalloidin probe (green). Nuclei were stained with Hoechst (blue). Scale bar: 30 µm. (**B**) Primary cultures of microglia were treated with α-syn or vehicle for 24 h, followed by treatment with JWH-133 (100 nM) or NMDA (15 µM) or the vehicle for another 24 h period. Then, cells were gently detached, and cell survival was assessed with a cell counter. % vs. basal vehicle is shown. Values are the mean ± SEM of five independent experiments performed in triplicates. Two-way ANOVA, followed by Tukey’s multiple comparison post hoc test, was used for statistical analysis (** *p* < 0.01, versus vehicle basal). (**C**) Proximity ligation assay (PLA) was performed in primary cultures of microglia treated or not with α-syn fibrils. Confocal microscopy images are shown (superimposed sections) in which heteromers appear as red clusters. Cell nuclei were stained with Hoechst (blue). Scale bar: 30 μm. Magnifications are shown on the right; white arrows indicate PLA dots. Quantification of the number of red dots/cells with dots (r) and of the percentage of cells presenting red dots is shown. Values are the mean ± SEM (*n* = 6). One-way ANOVA, followed by Tukey’s multiple comparison post hoc test, was used for statistical analysis (*** *p* < 0.001, versus vehicle). (**D**–**G**) Primary cultures of microglia treated (**E**,**G**) or not (**D**,**F**) with α-syn were stimulated with the selective antagonists (1 µM SR144528 for CB_2_R or 1 µM MK-801 for NMDAR), followed by agonist stimulation (100 nM JWH-133 for CB_2_R and/or 15 µM NMDA for NMDAR) and cAMP levels (**D**,**E**), and MAPK phosphorylation signals were measured (**F**,**G**). Values are the mean ± SEM of six different experiments. One-way ANOVA, followed by Tukey’s multiple comparison post hoc test, were used for statistical analysis (* *p* < 0.05, ** *p* < 0.01, *** *p* < 0.001; versus forskolin in cAMP assay or versus basal in MAPK phosphorylation).

To study how α-syn fibrils affect CB_2_R-NMDAR complexes in a more physiological context, we next studied the effects of α-syn fibrils on this heteromer in microglial cells, as they are key players in the inflammation process, where CB_2_R has an important role, as mentioned above. Therefore, rat microglia primary cultures were treated with α-syn fibrils. Immunocytochemistry with an anti-human alpha synuclein antibody was used to demonstrate that microglial cells are able to take up α-syn fibrils from the extracellular space, as a high fluorescent signal was detected in cells treated with fibrils compared to vehicle-treated cells (Figure 4A).

The literature shows that α-syn fibrils decrease cell viability in primary cultures of neurons [31]. In contrast, cell survival assays showed that α-syn fibrils do not significantly affect the viability of microglia (Figure 4B). Treatment with CB_2_R agonist JWH-133 did not induce any change in cell survival, while treatment with NMDA decreased cell viability (Figure 4B).

To study heteromerization of CB_2_R and NMDAR in microglial cells, a proximity ligation assay (PLA) was performed in primary cultures of rat microglia. This technique allows the visualization of receptor–receptor complexes as red fluorescent dots under the microscope. The presence of red dots around cell nuclei was detected in these cells, indicating that CB_2_R and NMDAR are able to form heteromeric complexes in primary cultures of microglia, as previously described [30]. Microglia cells showed around 40 dots/cell, with 100% of the cells showing fluorescent dots, while treatment with α-syn fibrils decreased PLA signal to 8 dots/cell with dots and only 80% of the cells presenting red dots (Figure 4C). PLA negative control, in which one of the antibodies is omitted, resulted in two dots/cell with dots and only 30% of the cells showing signal (Figure 4C). Thus, α-syn fibrils treatment decreases the formation of complexes between CB_2_R and NMDAR.

Once we had determined how α-syn fibrils affected CB_2_R-NMDAR heteromerization, we aimed to address how these fibrils affect the functionality of the complex in microglia cells. When intracellular cAMP accumulation was analyzed, neither CB_2_R nor NMDAR agonists produced a significant signal (Figure 4D). However, upon α-syn fibrils treatment, a significant decrease in cAMP levels previously increased with forskolin was observed, both with CB_2_R agonist JWH-133 alone or in combination with NMDA (Figure 4E), indicating that α-syn fibrils treatment increases CB_2_R signaling through the G-protein-dependent pathway in microglial cells. These results agree with those from Navarro and colleagues, where they show that JWH-133 barely induces a cAMP decrease in resting microglial cells, while this signal is significantly increased upon microglial activation [21]. Furthermore, JWH-133 signal was blocked not only by CB_2_R antagonist SR144528, but also by NMDAR antagonist MK-801 (Figure 4D,E), indicating that there is a cross-antagonism effect from NMDAR to CB_2_R, as observed in HEK-293T cells.

Next, we examined ERK1/2 phosphorylation in primary cultures of microglia by the alpha-screen method. When cells were stimulated with CB_2_R agonist JWH-133, a significant ERK1/2 phosphorylation was detected, while treatment with NMDA did not induce activation of this pathway (Figure 4F). Co-treatment with both CB_2_R and NMDAR agonists together abolished the previously observed signal (Figure 4F), indicating that NMDAR activation blunts CB_2_R-mediated signaling in the MAPK pathway. Moreover, the signal observed upon JWH-133 treatment was blocked not only by CB_2_R antagonist SR144528, but also by NMDAR antagonist MK-801 (Figure 4F), indicating that also in this signaling pathway there is a cross-antagonism effect from NMDAR to CB_2_R. When microglial cells were treated with α-syn fibrils, surprisingly, JWH-133 signal was diminished compared to control cells (Figure 4G), suggesting that α-syn fibrils treatment decreases CB_2_R signaling through the MAPK pathway in microglial cells, in contrast to the effect observed in the cAMP pathway.

### 2.6. CB_2_R Activation Counteracts the Detrimental Phenotype of Microglia Activation Induced by α-Syn Fibrils

**Figure 5:**
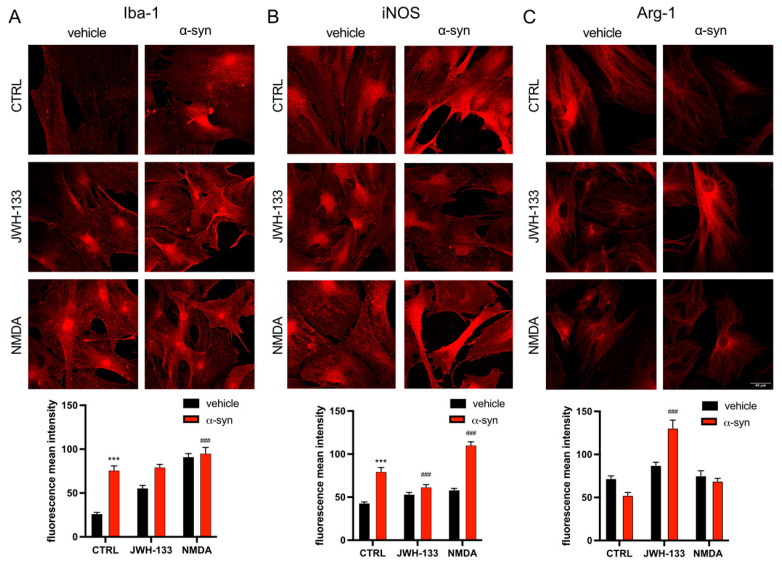
Analysis of the effects of α-syn fibrils on microglia polarization. Microglial markers in primary cultures treated with α-syn fibrils. Primary cultures of microglia were treated for 48 h with the specific agonist for CB_2_R, JWH-133 (100 nM) or for NMDAR, NMDA (15 µM) in the presence of α-syn fibrils. Immunocytochemical assays were performed using primary antibodies that detect either the Iba-1 microglial activation marker (**A**), the iNOS M1 marker (**B**), or the Arg-1 M2 marker (**C**), followed by a Cy3-conjugated secondary antibody (red). Fluorescence was quantified using Fiji program (raw fluorescence measurements are shown). Representative images for all conditions are shown. Scale bar 40 μm. Two-way ANOVA followed by Tukey’s multiple comparison post hoc test were used for statistical analysis (*** *p* < 0.001; versus vehicle control, ^###^ *p* < 0.001; versus α-syn control).

Finally, we studied the effect of α-syn fibrils on microglial polarization to either the detrimental M1 phenotype or the neuroprotective M2 phenotype, and if CB_2_R or NMDAR activation could counteract these effects. Therefore, primary cultures of microglia treated with α-syn fibrils and CB_2_R or NMDAR agonists were subjected to immunocytochemistry with antibodies for either Ionized calcium-binding adaptor molecule 1 (Iba-1) as a general indicator of microglial activation, Inducible Nitric Oxide Synthase (iNOS) as a marker of M1 phenotype, or Arginase-1 (Arg-1) as a marker of M2 phenotype. When microglial cells were treated with α-syn fibrils, Iba-1 signal significantly increased, and this increase was not modified upon treatment with neither CB_2_R agonist JWH-133 nor with NMDA (Figure 5A). Interestingly, treatment with NMDA alone also increased Iba-1 signal (Figure 5A). As expected, α-syn fibrils also increased iNOS signal, and, interestingly, this effect was counteracted by CB_2_R but not by NMDAR agonism (Figure 5B), which indicates that CB_2_R but not NMDAR activation can alleviate the polarization of microglia to the activated M1 phenotype. Finally, when analyzing Arg-1 signal, we could observe that it was slightly decreased by α-syn fibrils treatment (Figure 5C). However, treatment with CB_2_R agonist JWH-133 significantly increased Arg-1 signal, an effect that was not observed upon NMDA treatment (Figure 5C), reinforcing the idea that cannabinoids have a neuroprotective effect by not only decreasing the polarization of microglia to M1 phenotype, but also by increasing the transition to M2 phenotype.

## 3. Discussion

The interest in the use of cannabinoids as therapeutic agents is increasing, as new evidence appears showing the beneficial effects of these compounds in different pathologies [47]. Cannabinoids act mainly on cannabinoid receptors 1 and 2, which together with endocannabinoids and with the enzymes that synthetize and degrade these compounds form the endocannabinoid system. Parkinson’s disease is associated with alterations in the endocannabinoid system, such as changes in the levels of endocannabinoids in PD patients [48,49] and in rodents that overexpress α-synuclein [50]. Alterations in the expression of CB_1_R [51] and CB_2_R have been described both in patients [23,52] and animal models [23,24,25,26,53] of the disease.

These findings, together with the fact that NMDA receptors are also altered in Parkinson’s disease [36,38,39], led us to wonder if the complexes formed by CB_2_ and NMDA receptors could have a role in this pathology. First, we aimed to determine if α-syn fibrils altered receptor heteromerization, and we observed that α-syn fibrils increased the BRET signal in HEK-293T cells. This result apparently indicates that there is an increased heteromerization between CB_2_R and NMDAR induced by α-syn. However, PLAs, which allow us to detect heteromers in situ in native tissue, show that α-syn fibrils treatment significantly reduces the number of CB_2_R-NMDAR heteromers per cell in primary cultures of microglia, as previously observed for CB_1_R [31]. Hence, α-syn fibrils also affect heteromerization between CB_2_R and NMDAR. PLA and BRET results are, in principle, contradictory. Nevertheless, the increase in BRET signal observed upon α-syn treatment could be due to conformational changes within the CB_2_R-NMDAR complexes that cause a reorganization of the complex. This may decrease the distance between the Rluc and YFP proteins fused to the receptors, inducing a higher energy transfer between them.

When analyzing how the affinity of ligands for the orthosteric site of CB_2_R is affected in an in vitro PD model, α-synuclein preformed fibrils did not modify the affinity of the CB_2_R agonist JWH-133 for CB_2_R in HEK-293T cells expressing either CB_2_R alone or CB_2_R-NMDAR complexes. Hence, our observations showing that α-syn fibrils impair the signaling of CB_2_R, either when expressed alone or in combination with NMDAR, when analyzing intracellular cAMP accumulation, ERK1/2 phosphorylation, and β-arrestin II recruitment in HEK-293T cells, are not due to a decrease in the affinity of JWH-133 for CB_2_R. This reduction in cannabinoid receptor signaling upon α-syn fibrils incubation was previously detected for CB_1_R [31].

When analyzing Ca^2+^ signaling, although increased Ca^2+^ intracellular levels have been described upon exposure of neurons to α-syn oligomers in striatal slices [54], we observed a decrease in Ca^2+^ signaling in HEK-293T cells expressing CB_2_R-NMDAR complexes upon α-syn treatment. These results are intriguing but could be explained by the fact that HEK-293T cells lack many of the proteins present in neurons, thus the mechanisms controlling Ca^2+^ release in neurons are not present in HEK-293T cells. The most interesting observation when analyzing this pathway is the fact that the activation of cannabinoid receptor 2 impairs NMDAR signal, as was described for the CB_1_R-NMDAR complex [31], and which highlights the dual benefits of cannabinoids: on one hand they provide the well-known anti-inflammatory effects, and on the other hand they provide further benefit by decreasing NMDAR-induced excitotoxicity. Indeed, the use of NMDA receptor antagonist has proved to be effective for the treatment of PD symptoms [55,56]. There is evidence that NMDA receptor antagonism effectively improves motor symptoms in rodent PD models [57,58,59,60]. Selective NR2B antagonists, such as ifenprodil and traxoprodil, have shown efficacy in reducing L-DOPA-induced dyskinesia in both rodent models and MPTP-lesioned primates [61,62]. Also, NR2B antagonists have shown promising results in combination therapy approaches. For instance, the co-administration of radiprodil with the A_2A_R antagonist tozadenant resulted in enhanced motor performance in 6-OHDA-lesioned rats [63].

Parkinson’s disease is known to present neuroinflammation, as activated microglia has been found in the brains of PD patients [16,64]. Moreover, aggregated or mutated α-syn has been shown to induce microglial activation, which contributes to disease progression [13,65,66,67,68]. In the CNS, the expression of CB_2_R is higher in glia than in neurons [69], being upregulated in microglia under inflammatory conditions in vivo [25,26,70,71,72] and in vitro [21]. While CB_2_R expression in the healthy brain is relatively low, both preclinical animal models of neurodegenerative diseases and brain tissue of PD patients present an elevated CB_2_R expression in microglia [23]. In line with these findings, we have observed that in microglia treated with α-syn fibrils, cAMP response to CB_2_R agonist is increased compared to the control microglia, indicating that CB_2_R is able to elicit a stronger signal in the presence of α-syn fibrils in microglial cells. These results differ from those observed in HEK-293T cells, as microglial activation is strong enough to overcome the decrease in the signaling of CB_2_R upon α-syn fibrils’ incubation in HEK-293T cells via Gi protein. This differential effect in HEK-293T cells vs. microglial cells could be due to the increase in CB_2_R expression in activated vs. resting microglia, and also due to the different natures of both cell types, as HEK-293T cells derive from the kidney, and may not have the same signaling machinery as microglial cells. However, this stronger CB_2_R activation upon α-syn fibrils’ incubation was not observed when analyzing ERK1/2 phosphorylation in microglia. An interesting phenomenon observed when analyzing the functionality of the complex in microglial cells, that was also observed in HEK-293T cells, is the ability of NMDAR antagonist to block JWH-133-induced signal, namely cross-antagonism, a common print observed in GPCR heteromers. Likely, when MK-801 binds to NMDAR and the channel is blocked, NMDAR acquires a more rigid conformation. This rigidity could be transmitted to the other receptor in the complex, in this case CB_2_R, hampering the opening of transmembrane domain 6 and thus the binding of CB_2_R to the G protein.

Activated microglia can acquire a range of different phenotypes, including the proinflammatory M1 and the anti-inflammatory M2 opposed phenotypes. α-synuclein has been described to polarize activated microglia towards the detrimental M1 phenotype [65,73,74,75,76,77,78,79], and M1 microglia has been found in the MPTP model of PD [80,81,82,83]. Furthermore, CSF and serum of PD patients show high levels of proinflammatory markers [84]. On the contrary, microglia has been described to participate in the clearance of aggregated α-synuclein [85,86], therefore delaying the progression of the diseases. Specifically, α-synuclein clearance via M2 microglia polarization has been shown in experimental and human Parkinsonian disorder [87]. When we analyzed microglial activation markers, we observed that α-syn fibrils indeed promoted microglial polarization towards M1 phenotype, an effect that was counteracted by CB_2_R activation, but not by NMDAR activation. Moreover, activation of CB_2_R increased polarization to the protective M2 phenotype, an effect that was not observed upon NMDAR activation. These results point to a neuroprotective effect of CB_2_R activation in PD, as it was able to rescue the detrimental effects of α-syn fibrils in microglia. In line with these results, Mecha et al. demonstrated that CB_2_R expression in microglia is necessary to achieve M2 polarization [88]. Other studies have found increased expression of anti-inflammatory cytokines following CB_2_R-specific activation [89,90,91].

Altogether, our results point to an anti-inflammatory and thus neuroprotective effect of CB_2_R activation that could delay the progression of the disease by counteracting the detrimental effects of oligomeric α-synuclein. Previous studies support the role of CB_2_R as a therapeutic target for the treatment of PD, where CB_2_R activation resulted in reduced neuroinflammation, prevention of glial-derived neurotoxic mediator production, blood–brain barrier (BBB) leakage and peripheral immune cell infiltration, decreased death of dopaminergic neurons, and alleviation of motor impairments in different animal models of PD [23,27,92,93]. CB_2_R activation also promoted a reduction in the formation of alpha-synuclein aggregates in a rat model of nigral synucleinopathy [94]. Moreover, the absence of CB_2_R impaired motor abilities exacerbated the loss of dopaminergic neurons, and induced activation of the IL-1β pathway in the MPTP-induced mouse model of PD [93]. The lack of CB_2_R also resulted in an elevated α-synuclein-induced microglial synaptic pruning [95].

Nowadays, there is no cure for PD, and no neuroprotective therapy to prevent neuronal loss seems to be available. The most common strategy for the pharmacological treatment of PD tries to compensate for the loss of striatal dopamine by using the dopamine precursor L-DOPA. However, the use of L-DOPA produces secondary undesired effects known as dyskinesias, consisting of abnormal involuntary movements [12,96]. The study by Rentsch et al. demonstrates that the CB_2_R agonist HU-308 was able to reduce levodopa-induced-dyskinesia in mice [97], which demonstrates that cannabinoids could not only address PD pathological hallmarks, but also those derived from the use of L-DOPA. As mentioned above, using NMDAR antagonist has shown benefits in PD treatment.

One of the current lines of research to find new PD therapies focuses on α-synuclein, both in immunotherapy with the use of antibodies directed against α-synuclein and in the use of inhibitors of α-synuclein aggregation. Some clinical trials have already reached phase II, but these drugs are not in the market yet [7]. We propose the use of cannabinoids as new therapy for the treatment of PD. Cannabinoid action on CB_2_R could prevent neuronal death and promote polarization of microglia to the neuroprotective M2 phenotype. Indeed, the endocannabinoids, 2-arachidonoyl glycerol and anandamide, have antiparkinsonian properties; 2-AG is neuroprotective, and anandamide provides symptomatic relief [28]. Furthermore, cannabinoids have shown to be effective in decreasing axonal transport of α-syn fibrils [98]. Thus, the potential of cannabinoids for the treatment of PD arises as a promising new pathway that should be further explored.

## 4. Materials and Methods

### 4.1. Drugs

JWH-133 (#1343), N-Methyl-D-aspartate (NMDA) (#0114), SR 144528 (#5039), (+)-MK 801 maleate (#0924), and zardaverine (#1046) were purchased from Tocris (Bristol, UK). Forskolin (FK) (#HY-15371/CS-1454) was purchased from MedChemExpress (Sollentuna, Sweden). The CB_2_R agonist 3-[[4-[2-tert-butyl-1-(tetrahydropyran-4-ylmethyl) benzimidazol-5-yl]sulfonyl-2-pyridyl]oxy]propan-1-amine (CM157) conjugated to a fluorescent probe was developed in collaboration with Cisbio Assays (Codolet, France) (see [99]).

### 4.2. Cell Culture and Transient Transfection

HEK-293T cells (lot #612968, acquired from the American Type Culture Collection, Manassas, VA, USA) at passage 8–12 were grown in Dulbecco’s modified Eagle’s medium (DMEM) (15-013-CV, Corning, NY, USA) supplemented with 2 mM L-glutamine, 100 U/mL penicillin/streptomycin, MEM Non-Essential Amino Acids Solution (1/100), and 5% (*v*/*v*) heat inactivated fetal bovine serum (FBS) (Invitrogen, Paisley, Scotland, UK). Cells were maintained in a humid atmosphere of 5% CO_2_ at 37 °C. Briefly, HEK-293T cells growing in six-well dishes or in 25 cm^2^ flasks were transiently transfected using the PEI (PolyEthylenImine, Sigma-Aldrich, St. Louis, MO, USA) method. Cells were incubated for 4 h with the corresponding cDNAs together with PEI (5.47 mM in nitrogen residues) and 150 mM NaCl in a serum-starved medium. Then, the medium was replaced by a fresh complete culture medium, and cells were incubated for 48 h before experimental procedures.

To prepare primary microglial cultures, brain was removed from 2-day-old Sprague Dawley rats. Brains were dissected, carefully stripped off the meninges and digested with 0.25% trypsin for 20 min at 37 °C. Trypsinization was stopped by washing the tissue. Cells were brought to a cell suspension by passage through 0.9 mm and 0.5 mm needles, followed by passage through a 100 μm pore mesh. Glial cells were resuspended in medium and seeded at a density of 1 × 10^6^ cells/mL in 6-well dishes for cyclic adenylic acid (cAMP) assays, in 12-well dishes with coverslips coated with poly-D-lysine (A38904-01, Gibco, Paisley, Scotland, UK) for in situ proximity ligation assays and in 96-well plates for mitogen-activated protein kinase (MAPK) activation experiments. Cell counting was assessed using trypan blue and a Countess II FL automated cell counter (ThermoFisher Scientific-Life Technologies, Waltham, MA, USA). Cultures were grown in DMEM (15-013-CV, Corning, NY, USA) supplemented with 2 mM L-glutamine, 100 U/mL penicillin/streptomycin, MEM non-essential amino acids preparation (1/100), and 5% (*v*/*v*) heat-inactivated fetal bovine serum (FBS) (Invitrogen, Paisley, Scotland, UK), and maintained at 37 °C in humidified 5% CO_2_ atmosphere and, unless otherwise stated, medium was replaced once a week. Purity of these cultures was tested, and 95% of the cells were positive for Iba-1 staining.

### 4.3. Fusion Proteins and Expression Vectors

The human cDNAs for the CB_2_ and GHSR_1A_ receptors and NR1A and NR2B NMDAR subunits cloned in pcDNA3.1 were amplified without their stop codons using sense and antisense primers. The primers harbored either unique BamHI and KpnI sites for CB_2_R, EcoRI and KpnI sites for GHSR_1A_ or BamHI and HindIII sites for NR1A. The fragments were subcloned to be in frame with an enhanced yellow fluorescent protein (pEYFP-N1; Clontech, Heidelberg, Germany) or an Rluc (pRluc-N1; PerkinElmer, Waltham, MA, USA) on the C-terminal end of the receptor to produce NR1A-Rluc, CB_2_R–YFP, and GHSR_1A_-YFP fusion proteins. The plasmid encoding for the SNAP-tagged human CB_2_R used HTRF assays was obtained from Cisbio Bioassays (PSNAP-CB_2_, Cisbio Assays, Codolet, France).

### 4.4. α-Synuclein Fibrils Treatment

HEK-293T or primary glial cell cultures were treated for 48 h with recombinant human α-synuclein fibrils obtained by sonication, at a final concentration of 10 µg/L. Fibrils were prepared as previously described [100,101].

### 4.5. Immunofluorescence Studies

HEK-293T or microglial cells growing on glass coverslips were fixed in 4% paraformaldehyde for 15 min and then washed twice with PBS containing 20 mM glycine before permeabilization with the same buffer containing 0.2% Triton X-100 (5 min incubation). Cells were treated for 1 h with PBS containing 1% bovine serum albumin. To detect the expression of NR1-Rluc, HEK-293T cells were labeled with a mouse anti-Rluc antibody (1/100; MAB4400, Millipore, Burlington, MA, USA) and subsequently treated with Cy3-conjugated anti-mouse IgG secondary antibody (1/200; 715-166-150; Jackson ImmunoResearch, West Grove, PA, USA) (1 h each). The expression of CB_2_R-YFP was detected by the YFP’s own fluorescence.

The presence of α-syn fibrils was detected with a mouse monoclonal anti-human α-synuclein antibody (1/300; ab1903, Abcam, Cambridge, UK), followed by incubation with a Cy3-conjugated anti-mouse IgG secondary antibody (1/200; 715-166-150; Jackson ImmunoResearch, West Grove, PA, USA) (1 h each). Phalloidin was detected with an AF488 pre-stained anti-phalloidin probe (1/200; A12379, ThermoFisher, Waltham, MA, USA).

For M1/M2 assays, microglial cells were labeled with either a goat anti-Iba1 (1/100; ab107159; Abcam, Cambridge, UK), a mouse anti-iNOS (1/100; NOS2 [C-11]: sc-7271; SCB, Dallas, TX, USA), or a mouse anti-arginase I (1/100; 610708; BD Biosciences, Franklin Lakes, NJ, USA) antibody, and subsequently treated with Cy3-conjugated anti-goat (1/200; 705-165-147; Jackson ImmunoResearch, West Grove, PA, USA [red]) or anti-mouse (1/200; 715-166-150; Jackson ImmunoResearch, West Grove, PA, USA [red]) IgG secondary antibodies (1 h each).

Nuclei were stained with Hoechst 33342 (1/100 from stock 1 mg/mL; ThermoFisher). The samples were washed several times and mounted on glass slides with Shandon^TM^ Immu-Mount^TM^ (9990402; ThermoFisher, Waltham, MA, USA). Samples were observed under a Zeiss 880 confocal microscope (Carl Zeiss, Oberkochen, Germany) equipped with an apochromatic 63× oil-immersion objective (N.A. 1.4), and with 405 nm, 488 nm, and 561 nm laser lines.

### 4.6. Homogeneous Competition Binding Assays

HEK-293T cells growing in 25 cm^2^ flasks were transiently transfected with either SNAP-CB_2_R or SNAP-CB_2_R together with NR1 plus NR2B subunits of NMDAR. For SNAP protein labeling, 48 h after transfection, cell culture medium was removed and 100 nM SNAP-Lumi4-Tb (SSNPTBC, Cisbio Assays, Codolet, France) diluted in Tag-lite Buffer (TLB, Cisbio Assays, Codolet, France) was added to the cells and incubated for 1 h at 37 °C under 5% CO_2_ atmosphere. Cells were then washed with TLB to remove the excess of SNAP-Lumi4-Tb, detached with enzyme-free cell dissociation buffer, centrifuged for 5 min at 1500 rpm and resuspended in TLB. To perform competition binding assays 2500–3000 cells/well were placed in white opaque 384-well plates. Then, 20 nM fluorophore-conjugated CB_2_R ligand (labeled CM157) diluted in TLB was added to the cells, followed by increasing concentrations (0–10 μM range) of unlabeled JWH-133. Plates were then incubated for 2 h at room temperature before signal detection. Homogeneous time-resolved fluorescence energy transfer (HTRF) was detected using a PHERAstar Flagship microplate reader (Perkin-Elmer, Waltham, MA, USA) equipped with a FRET optic module, allowing donor excitation at 337 nm and signal collection at both 665 and 620 nm.

### 4.7. Bioluminescence Resonance Energy Transfer (BRET) Assays

HEK-293T cells growing in six-well plates were transiently co-transfected with a constant amount of cDNA encoding for NR1A fused to Renilla luciferase (NR1A-Rluc), with a constant amount of the cDNA encoding for NR2B, and with increasing amounts of cDNAs corresponding to CB_2_R or ghrelin receptor GHSR_1A_ fused to the yellow fluorescent protein (CB_2_-YFP, GHSR_1A_-YFP). Next, 48 h post-transfection cells were washed twice in quick succession in HBSS (137 mM NaCl; 5 mM KCl; 0.34 mM Na_2_HPO_4_; 0.44 mM KH_2_PO_4_; 1.26 mM CaCl_2_; 0.4 mM MgSO_4_; 0.5 mM MgCl_2_, and 10 mM HEPES, pH 7.4) supplemented with 0.1% glucose (*w*/*v*), detached by gently pipetting, and resuspended in the same buffer. To assess the number of cells per plate, we determined protein concentration using a Bradford assay kit (Bio-Rad, Munich, Germany) with bovine serum albumin dilutions as standards. To quantify YFP-fluorescence expression, we distributed the cells (20 μg protein) in 96-well microplates (black plates with a transparent bottom; Porvair, Leath-erhead, UK). Fluorescence was read using a Mithras LB 940 (Berthold, Bad Wildbad, Germany) equipped with a high-energy xenon flash lamp, using a 10 nm bandwidth excitation and emission filters at 485 and 530 nm, respectively. YFP-fluorescence expression was determined as the fluorescence of the sample minus the fluorescence of cells expressing protein–Rluc alone. For the BRET measurements, the equivalent of 20 μg of cell suspension was distributed in 96-well microplates (white plates; Porvair, Wrexham, UK), and we added 5 μM coelenterazine H (PJK GMBH, Kleinblittersdorf, Germany). Then, 1 min after coelenterazine H addition, the readings were collected using a Mithras LB 940 (Berthold, Bad Wildbad, Germany), which allowed the integration of the signals detected in the short-wavelength filter at 485 nm (440–500 nm) and the long-wavelength filter at 530 nm (510–590 nm). To quantify receptor–Rluc expression, we performed luminescence readings 10 min after addition of 5 μM coelenterazine H. The net BRET is defined as [(long-wavelength emission)/(short-wavelength emission)] − Cf, where Cf corresponds to [(long-wavelength emission)/(short-wavelength emission)] for the Rluc construct ex-pressed alone in the same experiment. The BRET curves were fitted assuming a single phase by a non-linear regression equation using the GraphPad Prism software version 10.2.1 (GraphPad, San Diego, CA, USA). BRET values are given as milli BRET units (mBU: 1000 × net BRET).

### 4.8. cAMP Level Determination

Two hours before initiating the experiment, HEK-293T or microglial cell-culture medium was exchanged to serum-starved DMEM or neurobasal medium, as corresponds. Then, cells were detached, resuspended in the serum-starved medium containing 50 µM zardaverine and plated in 384-well microplates (2500 cells/well), pretreated (15 min) with the corresponding antagonists—or vehicle—and stimulated with agonists (15 min), before adding 0.5 μM forskolin or vehicle. Readings were performed after 1 h incubation at 25 °C. Homogeneous time-resolved fluorescence energy transfer (HTRF) measures were obtained using the Lance Ultra cAMP kit (PerkinElmer, Waltham, MA, USA) [62]. Fluorescence at 665 nm was analyzed on a PHERAstar Flagship microplate reader equipped with an HTRF optical module (BMG Lab technologies, Offenburg, Germany).

### 4.9. Extracellular Signal-Regulated Kinase Phosphorylation Assays

HEK293T cells growing in 25 cm^2^ flasks were transfected with the cDNAs encoding for CB_2_R, for NR1A and for NR2B. Two to four hours before initiating the experiment, the culture medium was replaced by serum-starved DMEM. Cells were incubated at 37 °C with antagonists (15 min) or vehicle, followed by stimulation (7 min) with agonists. After that, the reaction was stopped by placing the cells on ice. Then, the cells were washed twice with cold PBS and lysed by the addition of ice-cold lysis buffer (50 mM Tris-HCl pH 7.4, 50 mM NaF, 150 mM NaCl, 45 mM glycerol-3-phosphate, 1% Triton X-100, 20 µM phenyl-arsine oxide, 0.4 mM NaVO4 and protease inhibitor mixture (MERK, St. Louis, MO, USA)). Cellular debris were removed by centrifugation at 13,000× *g* for 10 min at 4 °C, and protein concentration was adjusted to 1 mg/mL by the bicinchoninic acid method (ThermoFisher Scientific, Waltham, MA, USA), using a commercial bovine serum albumin dilution (BSA) (ThermoFisher Scientific, Waltham, MA, USA) for standardization. 6× Laemli SDS sample buffer (300 mM Tris-Base, 600 mM DTT, 40% glycerol (*v*/*v*), 0.012% bromophenol blue (*w*/*v*), and 12% SDS (*w*/*v*), pH = 6.8) was added to the samples and proteins were denatured by boiling at 100 °C for 5 min. ERK1/2 phosphorylation was determined by Western blot. Equivalent amounts of protein (20 μg) were subjected to electrophoresis (10% SDS-polyacrylamide gel) and transferred onto PVDF membranes (Immobilon-FL PVDF membrane, MERK, St. Louis, MO, USA) for 30 min using Trans-Blot Turbo system (Bio-Rad). Then, the membranes were blocked for 2 h at room temperature (constant shaking) with Odyssey Blocking Buffer (LI-COR Biosciences, Lincoln, NE, USA) and labeled with a mix of primary mouse anti-phospho-ERK 1/2 (1/2500, MERK, Ref. M8159) and rabbit anti-ERK 1/2 (1/40,000, MERK, Ref. M5670) antibodies overnight at 4 °C with shaking. Then, the membranes were washed three times with PBS containing 0.05% tween for 10 min and were subsequently incubated with a mix of IRDye 800 anti-mouse (1/10,000, MERK, Ref. 92632210) and IRDye 680 anti-rabbit (1/10,000, MERK, Ref. 926-68071) secondary antibodies for 2 h at room temperature, light protected. Membranes were washed three times with PBS-tween 0.05% for 10 min and once with PBS and left to dry. Bands were analyzed using Odyssey infrared scanner (LI-COR Biosciences). Band densities were quantified using Fiji software version 1.54f, and the level of phosphorylated ERK1/2 was normalized using the total ERK 1/2 protein band intensities. Results are represented as the percentage over basal (non-stimulated cells).

To determine extracellular signal-regulated kinase 1/2 (ERK1/2) phosphorylation in microglial primary cultures, cells were grown in 96-well plates. On the day of the experiment, the medium was replaced by serum-free medium 2 h before starting the experiment. Cells were pre-treated at 25 °C for 15 min with antagonists or vehicle, and stimulated for an additional 15 min with selective agonists. Cells were then washed twice with cold PBS before the addition of lysis buffer (a 15 min treatment). Afterward, 10 µL of each supernatant were placed in white ProxiPlate 384-well plates and ERK 1/2 phosphorylation was determined using an AlphaScreen^®^SureFire^®^ kit (Perkin Elmer, Waltham, MA, USA), following the instructions of the supplier, and readings were collected using an EnSpire^®^ Multimode Plate Reader (PerkinElmer, Waltham, MA, USA). The value of reference (100%) was the value achieved in the absence of any treatment (basal). The effect of ligands was given in percentage with respect to the basal value.

### 4.10. Detection of Cytoplasmic Calcium Levels

HEK-293T cells were co-transfected with the cDNA for the corresponding receptors (see figure legend) together with the cDNA for the GCaMP6 calcium sensor [46]. Then, 48 h after transfection, HEK-293T cells were detached using Mg^2+^-free Locke’s buffer (154 mM NaCl, 5.6 mM KCl, 3.6 mM NaHCO_3_, 2.3 mM CaCl_2_, 5.6 mM glucose, 5 mM HEPES, 10 μM glycine, pH 7.4), centrifuged for 5 min at 3200 rpm, and resuspended in the same buffer. Protein concentration was quantified by using the Bradford assay kit (Bio-Rad, Munich, Germany). To measure Ca^2+^ mobilization, cells (40 µg of protein) were distributed in 96-well microplates (black plates with a transparent bottom; Porvair, Leatherhead, UK) and were incubated for 10 min with antagonists when indicated. Fluorescence readings were performed right after the addition of agonists. Fluorescence emission intensity due to GCaMP6 was recorded at 515 nm upon excitation at 488 nm on the EnSpire^®^ Multimode Plate Reader for 300 s every 5 s at 100 flashes per well.

### 4.11. β-Arrestin II Recruitment

To determine β-arrestin recruitment, BRET experiments were performed in HEK-293T cells 48 h after transfection with the cDNA for either CB_2_R-YFP, or CB_2_R-YFP and NR1 and NR2B subunits of NMDAR, together with cDNA corresponding to β-arrestin II-Rluc. Cells (20 μg protein) were distributed in 96-well microplates (Corning 3600, white plates with white bottom, Corning, NY, USA) and incubated with antagonists for 10 min prior to the addition of agonists. Immediately after, 5 μM coelenterazine H (Molecular Probes, Eugene, OR, USA) was added, and 7 min after coelenterazine H addition, BRET readings corresponding to β-arrestin II-Rluc and receptor-YFP were quantified. The readings were collected using a Mithras LB 940 (Berthold Technologies, Bad Wildbad, Germany) that allows the integration of the signals detected in the short-wavelength filter at 485 nm and the long-wavelength filter at 530 nm. To quantify protein–Rluc expression, luminescence readings were performed 10 min after the addition of 5 μM coelenterazine H.

### 4.12. Cell Viability

Cell viability assay is based on the principle that living cells maintain intact cell membranes that exclude certain dyes, like trypan blue. To quantify the percentage of living cells, DIV 14 primary cultures of microglial cells growing in six-well plates were treated with 10 µg/L α-syn or vehicle on DIV 15, cells were treated either with 100 nM JWH-133, 15 µM NMDA, or vehicle for another 24 h. HEK-293T cells growing in six-well plates were treated with increasing concentrations of α-syn or vehicle for 48 h.

On the day of the experiment, cells were gently detached and mixed with an equal volume of trypan blue (0.4%) (trypan blue solution, T8154, Sigma Aldrich (St. Louis, MO, USA)). Cells (%) were counted in a Countess II FL automated cell counter (ThermoFisher Scientific, Waltham, MA, USA).

### 4.13. In Situ and In Vitro Proximity Ligation Assay (PLA)

The proximity ligation assay allows the detection of molecular interactions between two endogenous proteins ex vivo. PLA requires both receptors to be sufficiently close (<16 nm) to allow double-strand formation of the complementary DNA probes conjugated to the antibodies. Using the PLA, the heteromerization of NR1 subunits of NMDAR with CB_2_ receptors was detected in situ in primary cultures of microglial cells.

The presence/absence of receptor–receptor molecular interactions in the samples was detected using the Duolink II In Situ PLA Detection Kit (developed by Olink Bioscience, Uppsala, Sweden; and now distributed by Sigma-Aldrich (St. Louis, MO, USA) as Duolink^®^ using PLA^®^ Technology). The PLA probes were obtained after conjugation of the primary anti-NR1 antibody (ab52177, Abcam, Cambridge, UK) to a MINUS oligonucleotide (DUO92010, Sigma-Aldrich, St. Louis, MO, USA), and the anti-CB_2_R (ab230791, Abcam, Cambridge, UK) antibodies to a PLUS oligonucleotide (DUO92009, Sigma-Aldrich, St. Louis, MO, USA). The specificity of antibodies was tested in non-transfected HEK-293T cells. Microglial cells growing in glass coverslips were fixed in 4% paraformaldehyde for 15 min and then washed twice with PBS containing 20 mM glycine before permeabilization with PBS-glycine containing 0.2% Triton X-100 for 5 min. After permeabilization, samples were washed in PBS at room temperature and incubated in a preheated humidity chamber for 1 h at 37 °C with the blocking solution provided in the PLA kit. Then, samples were incubated overnight with the PLA probe-linked antibodies (1/100 dilution for all antibodies) at 4 °C. After washing, samples were incubated with the ligation solution for 1 h and then washed and subsequently incubated with the amplification solution for 100 min (both steps at 37 °C in a humid chamber). Nuclei were stained with Hoechst 33342 (1/100 from stock 1 mg/mL; ThermoFisher, Waltham, MA, USA). The samples were washed several times and mounted on glass slides with Shandon^TM^ Immu-Mount^TM^ (9990402; ThermoFisher, Waltham, MA, USA). Negative controls were performed by omitting the anti-CB_2_R-PLUS antibody. Samples were observed under a Zeiss 880 confocal microscope (Carl Zeiss, Oberkochen, Germany) equipped with an apochromatic 63× oil-immersion objective (N.A. 1.4), and with 405 and 561 nm laser lines. For each field of view, a stack of two channels (one per staining) and 9 Z planes with a step size of 0.5 µm were acquired. The ratio r (number of red spots/cell) was determined on the maximum projection of each image stack using the Duolink Image tool software (DUO90806, version 1.0.1.2, Sigma-Aldrich, St. Louis, MO, USA).

### 4.14. Data Analysis

Data, expressed as the mean ± SEM, were obtained from at least five independent experiments. Data comparisons were analyzed by one-way ANOVA or two-way ANOVA, followed by Bonferroni’s post hoc test. The normality of populations and homogeneity of variances were tested before the ANOVA. For the two-way ANOVA, factor 1 was the presence of α-syn, and factor 2 was the drug treatment. Statistical analysis was undertaken only when each group size was at least *n* = 5, n being the number of independent variables (technical replicates were not treated as independent variables). Differences were considered significant when *p* ≤ 0.05. Statistical analyses were carried out with GraphPad Prism software version 10.2.1 (GraphPad, San Diego, CA, USA). Outliers’ tests were not used, and all data points (mean of replicates) were used for the analyses.

## Data Availability

Data can be obtained from the corresponding author upon reasonable request.

## Abbreviations

The following abbreviations are used in this manuscript:

6-OHDA	6-hydroxydopamine
Arg-1	Arginase-1
BBB	Blood–brain barrier
BRET	Bioluminiscence Resonance Energy Transfer
CB2R	Cannabinoid receptor 2
GHSR1A	Ghrelin receptor 1A
GPCRs	G protein-coupled receptors
HEK-293T	Human Embryonic Kidney 293T
HTRF	Homogeneous Time-Resolved FRET
Iba-1	Ionized calcium-binding adaptor molecule 1
iNOS	Inducible Nitric Oxide Synthase
L-DOPA	Levodopa
LPS	Lipopolysaccharide
mBU	Milli BRET units
MPTP	1-methyl-4-phenyl-1,2,3,6-tetrahydropyridine
NMDA	N-methyl-D-aspartate
NMDAR	NMDA receptor
PD	Parkinson’s disease
PLA	Proximity Ligation Assay
Rluc	Renilla luciferase
YFP	Yellow fluorescent protein
α-syn	Alpha synuclein
